# What’s important for recovery after a total knee replacement? A systematic review of mixed methods studies

**DOI:** 10.1007/s00402-023-05136-x

**Published:** 2023-12-09

**Authors:** Chetan Khatri, Imran Ahmed, Fatema Dhaif, Jeremy Rodrigues, Martin Underwood, Edward T. Davis, Paul Mitchell, Andrew Metcalfe

**Affiliations:** 1 Clinical Trials Unit, University of Warwick, Coventry, UK; 2 Royal Orthopaedic Hospital NHS Foundation Trust, Birmingham, UK; 3 Health Economics Bristol, Population Health Sciences, University of Bristol, Bristol, UK; 4 Clinical Sciences Research Laboratories, University Hospitals Coventry & Warwickshire, Clifford Bridge Road, Coventry, CV2 2DX UK

**Keywords:** Mixed methods review, Total knee replacement, Attributes, Discrete choice experiment

## Abstract

**Background:**

Understanding how patients perceive and prioritise various aspects of recovery following total knee replacement, including pain, function and return to activity, will help clinicians in pre-operative consultations by ensuring they effectively address patient concerns and managing their expectations.

**Aims:**

The aim of this study is to identify aspects of recovery that are important to people after a total knee replacement.

**Methods:**

Studies were identified from Medline, Embase, PsycInfo, Cochrane Library and Web of Science. This mixed methods review included all original study types (quantitative, qualitative, discrete choice experiments and mixed methods design). Reviews and non-peer-reviewed publications were excluded. Studies with participants (age ≥ 18 years) who had a primary TKR for osteoarthritis were included. Studies of people with unicompartmental knee, patella-femoral or revision knee replacement were excluded. Recovery attributes were extracted from individual papers and grouped into recovery themes.

**Results:**

A total of 23 studies with 8404 participants and 18 recovery themes were developed. The most frequently identified overarching theme was pain, followed by activities of daily living, mobility (walking), recreational activities, specific functional movements of the knee, use of walking aids, sexual activity and range of motion of the knee. Medical complications were an infrequently reported theme, however, was deemed to be high importance.

**Conclusions:**

Reducing pain, returning of activities and daily living and mobility are the three most frequently reported recovery domains for people after TKR. Clinicians should be aware of recovery themes, to ensure they are explored sufficiently when consenting for a TKR. Future research should aim to determine the relative importance of these attributes compared to each other.

** Review Registration:** https://www.crd.york.ac.uk/prospero/display_record.php?ID=CRD42021253699

**Supplementary Information:**

The online version contains supplementary material available at 10.1007/s00402-023-05136-x.

## Background

Osteoarthritis of the knee can result in pain, stiffness and disability. Total knee replacement (TKR) is a common procedure that aims to alleviate pain and improve function. Around 100,000 TKRs are performed annually in the United Kingdom [6, 52]. The number of knee replacements has risen every year over the past decade [28].

Whilst knee replacement surgery can improve quality of life by reducing pain and disability, the procedure is associated with risks. In the short term, these include infection and the risk of venous thromboembolism. In the longer term, the primary concerns for the patients may include (chronic) pain, stiffness and loss of independence. Surgeons may focus on failure of the components, such as loosening which may be related to infection. Even without these complications, around 10% of people can be dissatisfied with the outcome of their operation, despite a technically and radiologically satisfactory procedure [1, 2, 8, 17, 30, 50]. Risk factors for dissatisfaction include sex, primary diagnosis (osteoarthritis *vs.* inflammatory arthritis), co-existing back pain, history of depression, low pre-operative patient-reported outcome measures (PROMs) and wound healing complications [45, 53, 54]. Surgeons and researchers have sought to improve care by modifying pre-operative risk factors or improving intra-operative techniques to improve post-operative success [22]. However there is no granular insight into which domains of recovery define ‘success’, and which domains are important to those undergoing TKR.

There are numerous domains of recovery which may be important and can be measured. New interventions may produce differential improvement in early, or mid- or long-term outcomes, or may not have an effect at all. Randomised controlled trials often collect these domains as primary or secondary outcome measures. Having evidence on the importance (and relative weighting) of these recovery attributes may provide a standardised, evidence-based approach in interpretation of the overall package of outcomes which measure recovery.

A potential methodology to assess and give quantitative weighting is discrete choice experiments (DCEs) [51]. Here, hypothetical scenarios are presented to participants to choose between. Each scenario has a varying number of attributes, which may be domains such as pain or ability to walk. Each attribute has further detail known as levels, which may be moderate pain, some pain and no pain. Through repeated choice of scenarios with varying levels of the same, fixed attributes, estimations can be made to quantifiably rank attributes in terms of importance. The first step of identification of suitable attributes is through systematic review of the literature [25, 48].

For health researchers, understanding which recovery themes are important is pertinent when designing new interventions. Interventions should be targeted to improve outcomes in recovery domains that people find important.

Elucidating recovery preferences is applicable for clinicians as well. The common sense model [36] suggests that people choose what treatment they undergo based on what their expectations are for recovery after a given treatment. The National Institute for Health and Care Excellence suggests that these expectations and individual values ought to be taken into account during the selection of treatment offered [24]. Understanding opinions of the population they treat will help better inform them of their expected recovery. As unmet expectations influence post-operative outcomes and satisfaction [17], this forms an central aspect of informed consent for what is a major surgical procedure.

The aim of this review is to identify published data describing which recovery themes are important to people when considering a total knee replacement.

### Research question

What are the recovery attributes for people in their decision-making processes when considering a total knee replacement?

## Methods

A mixed methods systematic review of the literature was conducted and reported in accordance with the Preferred Reporting Items for Systematic Reviews and Meta-Analyses (PRISMA) statement [49]. The study protocol was predefined and registered in PROSPERO (https://www.crd.york.ac.uk/prospero/CRD42021253699).

A bespoke search strategy comprising index and free text terms was created with a university librarian (supplementary file 1). This was applied to EMBASE, Medline, Web of Science, Cochrane library and PsycInfo from inception to 11th of December 2021.

### Participant eligibility criteria

The inclusion criteria were studies that assess and analyse the importance of preferences of people in the post-operative recovery after a total knee replacement, regardless of the methods used. As this study focussed on the post-operative recovery, studies were excluded if they solely investigated factors related to the pre-operative period (e.g. referral pathways, time to receive information) or operation-related factors (e.g. seniority of surgeon), as these were felt to be out of the scope of this review, which was focussed on recovery after surgery.

The exclusion criteria were studies of people with unicompartmental knee replacement (partial knee replacement), patellofemoral replacement or revision total knee replacement.

### Study type eligibility criteria

This mixed methods study includes original studies that analysed preferences important to participants regardless of method used. This includes quantitative studies, qualitative studies, discrete choice experiments (DCEs), mixed methods design, ranking studies and best–worst scaling. Only English language studies were included.

Studies were excluded if they were systematic reviews, non-peer-reviewed articles (such as theses, news articles) or not having preference weights for recovery attributes.

### Data extraction

Electronic lists of study citations, including title and abstract authors, were imported into EndNote 20. Duplicates were removed and two authors (CK and IA) screened citations based on title and abstracts as per the eligibility criteria. If there was any doubt about inclusion criteria, the article proceeded to the next stage. Studies that potentially met the eligibility criteria had full text retrieved. Two authors (CK and IA) independently assessed each paper according to the edibility criteria. Where consensus was not reached, a third reviewer (AM) arbitrated.

Data for study characteristics extracted included year of publication, country, number of participants and design type.

The main variables of interest were recovery attributes and the frequency at which they were reported. Their relative ranking or importance as stated by the study authors was extracted. Where data were not presented numerically (for example, they were on a bar chart), authors were contacted for raw numerical data. In the absence of a response, two authors (CK and IA) estimated numerical data from charts by manual measurement as has been described in previous systematic review methodology [33]. Where data were not available, or mixed populations were present (e.g. total knee replacement and total hip replacement), authors were contacted up to three times for data. If no data on TKR-only populations were provided, the study was excluded.

Attributes were extracted, with their relevant importance and potential levels of measurement, as defined by their original study. Using principles from a thematic approach as described by Braun and Clarke, raw attributes were read and re-read to familiarise with data [9]. Attributes were considered first-order constructs and were categorised into second-order constructs (overarching themes). Two authors (CK and FD) independently assessed each attribute and placed into themes. If agreement was not achieved, a senior author arbitrated (AM).

### Quality assessment

Each study was appraised using the Mixed Methods Appraisal Tool (MMAT) [26]. This is a critical appraisal tool that has been specifically designed for the appraisal stage of a mixed methods systematic review. It permits to appraise the methodological quality of five categories of studies: qualitative research, randomised controlled trials, non-randomised studies, quantitative descriptive studies and mixed methods study. As such, it represents a unifying appraisal tool for the purpose of this study. As per the recommendation from the MMAT working group, excluding studies with low methodological quality is discouraged when using this tool. Two authors (CK and FD) independently assessed each included paper. Discrepancies were discussed and where consensus was not reached was discussed with a third author (IA).

## Results

**Fig. 1:**
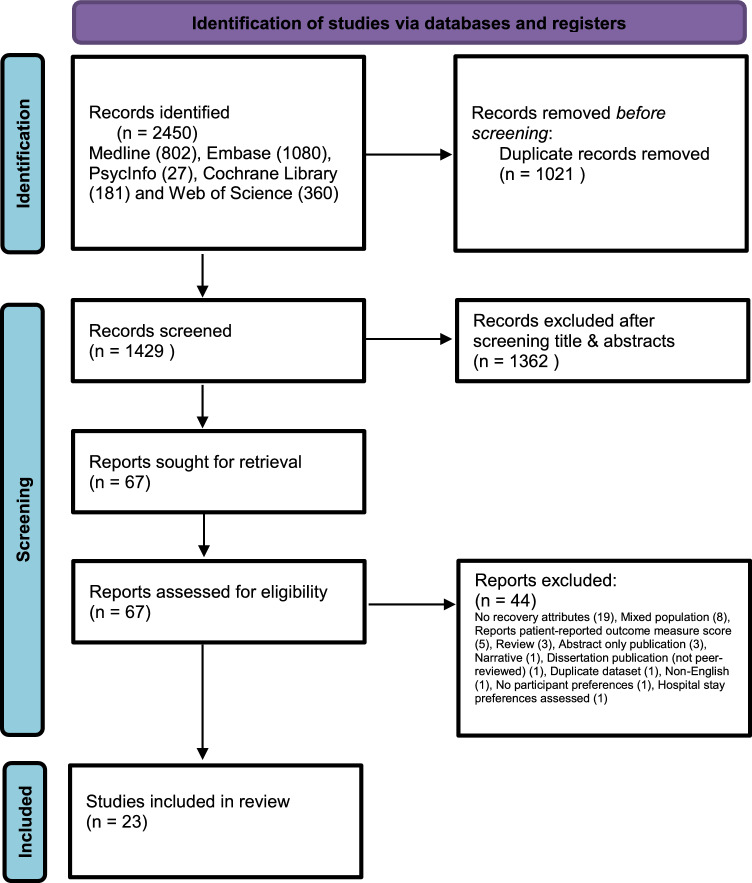
PRISMA flow diagram

This study identified 1429 individual citations, reviewed 67 full text publications and included 23 studies. A PRISMA flow chart is available via Fig. 1.

### Demographics

Papers were published from 1999 to 2020 from a wide geographical spread including North America *n* = 9, Europe *n* = 9, the Middle East *n* = 1, Asia *n* = 2 and Australia *n* = 2 (Table1). The total number of participants was 8404 of whom 4743 (56%) were female. Most studies were questionnaire surveys (*n* = 16), followed by interview-based studies (*n* = 4), mixed methods studies (*n* = 1), focus group (*n* = 1) and DCE (*n* = 1).

**Table 1:** Study characteristics

Author	Study type	Year	Country	MMAT^a^	*n*	Male (*n*)	Female (*n*)
Bin Sheeha [7]	Qualitative (focus group)	2020	Saudi Arabia	100	7	2	5
Cross [14]	Quantitative (questionnaire)	2009	Australia	80	78	39	39
Chan [10]	Quantitative (questionnaire)	2013	Australia	100	171	66	105
Cheow [11]	Quantitative (questionnaire)	2013	Singapore	80	105	22	83
De Achaval [16]	Quantitative (questionnaire)	2016	United States of America	100	236	82	154
Devers [18]	Quantitative (questionnaire)	2011	United States of America	80	122	n/a	n/a
Espinosa [21]	Quantitative (questionnaire)	2020	Spain	40	183	n/a	n/a
Harmsen [23]	Quantitative (questionnaire)	2019	The Netherlands	80	866	399	467
Hutyra [27]	Quantitative (DCE)	2020	United States of America	100	258	92	164
Jeffery [29]	Qualitative (interview)	2011	United Kingdom	100	58	10	18
Kwoh [35]	Quantitative (questionnaire)	2015	United States of America	80	799	290	509
Lewis [37]	Qualitative (interview)	2014	United States of America	100	14	7	7
Lingard [38]	Quantitative (questionnaire)	2006	United States of America	100	598	254	344
Macario [39]	Mixed methods	2003	United States of America	80	19	5	14
Mahdi [40]	Qualitative (interview)	2020	Sweden	100	44	16	28
Mannion [42]	Quantitative (questionnaire)	2009	Switzerland	80	112	34	78
Mavalankar [43]	Quantitative (questionnaire)	2019	India	60	74	26	48
McGrory [44]	Quantitative (questionnaire)	1999	United States of America	60	266	117	149
Muniesa [46]	Quantitative (Questionnaire)	2009	Spain	100	497	122	375
Noble [47]	Quantitative (questionnaire)	2012	United States of America	60	598	48	53
Scott [55]	Quantitative (questionnaire)	2012	United Kingdom	100	323	127	196
Smith [20]	Quantitative (questionnaire)	2015	United Kingdom	100	200	82	112
Wiering [57]	Quantitative (questionnaire)	2017	The Netherlands	100	2776	942	1834
			Total		8404	2743	4743

^a^MMAT represented as percentage of questions with answer ‘Yes’. Full details of MMAT assessment can be found in supplementary materials (Supplementary file 2)

### Quality assessment

The MMAT assessment of studies showed an overall high quality of study methodology and reporting with the majority of studies fulfilling the criteria for their respective study design (Table 2). Only one study has less than 50% of assessment, this was due to unclear reporting (supplementary file 2).

**Table 2:** Overarching themes

Overarching theme	Number of studies	Examples
Pain	16	Pain relief by operation
Activities of Daily Living	15	Improve ability to perform daily activities Improve ability to use public transportation or drive
Mobility (Walking)	13	Daily time spent walking Ability to walk as much as you wish
Recreational Activities and Sports	9	Improvement for leisure activities Improve ability to exercise/play sport
Specific Functional Movements	8	Improve ability to squat Improve ability to kneel
Use of Walking Aids	8	Remove need for crutch Walk 5 blocks without cane
Range of Motion of the Knee	8	Stiffness (lack of bending) Knee flexion
Sexual Activity	7	Improve sexual activity
Psychological Well-Being	5	Improve psychological well-being
Employment	4	Employed for monetary reimbursement
Forgottenness of Joint Replacement	3	Forgottenness of joint
Medical Complications	3	Serious complications
Timing of Recovery	2	Time to recovery
Cosmetic Appearance	2	Will the surgery change the way I look
Length of Stay	2	How long will I be in the hospital
Stability	1	Stability
Overall Experience	1	Overall recovery experience
Need for Physical Therapy	1	Am I going to need physical therapy

### Excluded studies

Five studies that used patient-reported outcome measurement (PROMs) instruments were excluded [5, 26, 38, 52, 55]. These studies used instruments to report satisfaction with recovery [5, 26, 38], predict residual symptoms [52] or to compare between providers [55]. They did not report preferences or importance of individual items.

### Attributes within studies

Most studies (17 out of 23) had a quantitative study design (Table 2). They posed items to participants, representing a mixture of pain, mobility and functional tasks (e.g. pain at night, going up and down stairs, kneeling). These items were mostly derived from commonly used PROM instruments such as the WOMAC, OKS, KSS and Hospital for Special Surgery (HSS) [5, 15, 41, 47]. In some studies, questions about function or activities of daily living were generated from the study authors; however, no further information was provided about the origin of these questions [43]. Participants were then asked to rank either the importance, relevance or concern for each of the items. Using a combination of these methods, the relative importance of each item was generated. During their development, many PROM instruments undergo such an iterative, relative importance ranking exercise. However, in these studies, the items posed were fixed, without room to introduce new attributes or opinions into the individual studies.

Four studies used qualitative designs (three interview, one focus group) to elicit issues. The first study conducted semi-structured interviews within the setting of focus groups to ask about reasons for satisfaction in a post-operative population [7]. Thematic analysis was used to derive attributes from interview transcripts which allowed attributes to be developed de novo. However, no importance or preferencing was provided per attribute. The second paper used interviews to delineate issues important to people with chronic pain after TKR [29]. Again, thematic analysis was used, where whilst allowing for attributes to be developed, no importance was applied to each developed attribute. This study only focussed on those who were dissatisfied, introducing bias into the issues that were developed and ranked. The third paper asked those who were dissatisfied with their knee arthroplasty reasons for their discontent [40]. The authors asked participants expectations of recovery in each domain identified, giving timelines for expected recovery in each domain. Again, as this was a cohort who were dissatisfied, this may introduce bias meaning the attributes are not generalisable to a wider population. The final paper developed a new PROM instrument, labelled the Patient’s Knee Implant Performance (PKIP) questionnaire, using a cognitive interview technique [37]. The attributes posed to participants were generated through a literature search, and these were presented to participants, who then commented on the relevance of each item with an aim to reduce to nine items that would form the final PKIP instrument.

### Themes

We developed 18 themes from attributes derived from individual studies (Table 1). A full detail of individual attributes and their ranking within each study can be found in supplementary file 3. The overarching theme cited by the most authors with a consistently high priority was pain, being mentioned in 16 studies: most studies grouped post-operative pain, without discriminating by character or timescale (early vs late), and a small number of studies differentiated by immediate post-operative pain, or timing of pain (day-time vs night-time). Of those studies reporting importance, it was often ranked as the most important attribute, or with a high proportion (> 80%) of participants ranking it as very important.

The second most frequent was activities of daily living, cited by 15 studies. This theme had the largest breadth, and number of attributes including going up and down stairs, using public transport and performing household chores/activities. Many of these attributes were derived from existing PROMs such as the WOMAC, Oxford Knee Score (OKS) and Knee Society Scoring system (KSS) scores [5, 47]. All these attributes had their relative importance reported, with most studies scoring this theme highly, with individual attributes ranked in the top five.

Mobility was commonly identified theme. People largely prioritising daily time spent walking. In studies that reported importance, it was highly valued, with the majority of studies reporting that > 90% of participants expecting recovery in walking and ranked it to be the most important. There were some studies which ranked walking to be of low priority [21, 47]. A theme related to this was the use of walking aids; however, the importance was felt not to be a priority, receiving low rankings in most studies overall (e.g. 20% expected not using a walking aid at 1 year [21, 57]). Specific movements such as the ability to squat and kneel were mentioned frequently, however, had varying levels of importance. One study ranked it as the most important [16], whilst others ranked low importance (19.4% of study cohort ranked as very important) [57].

There were attributes that had elements of crossover between themes. Examples of this included using public transport, which possibly reflects two themes: mobility (walking) and activities of daily living. In such scenarios, the attribute was placed into a particular theme based on the context of which it was presented to the participants within a study.

The next largest theme was taking part in recreational activities and sports. This included a wide variety of sports, including high function activities such as dancing and running. There were also generic attributes such as ‘improving ability to exercise’ [55]. The importance of these attributes was mixed, with attributes representing social activity and participation with others to be high (e.g. social activities were expected by 92.4% of participants [46]), in contrast, doing specific sports to be of low importance [47], demonstrating that results are likely to be context specific.

Sexual activity was mentioned in seven studies and had contrasting results with respect to importance. Whilst some studies reported low expectation of return of sexual function (0.5% expecting improvement [21]), other studies ranked it higher (mean score of 2.2, with 3.0 being very important [14]).

A less frequently mentioned theme was medical complications. Whilst this was only addressed in three studies, all studies ranked these highly in importance, with one study ranking it as the most important domain [27]. Employment was mentioned by five studies; however, almost all studies reported this is a low priority. Psychological well-being was mentioned in five studies, with three studies giving high importance to it (mean score of 2.3, 3.0 being very important [14]), but the other two studies giving low importance (one study reporting only 5% expected employment [46]).

Other themes that were infrequently mentioned (≤ 3 studies) included ability to forget the artificial joint, timing of recovery, cosmetic appearance, length of stay, stability, overall experience and need for physical therapy.

## Discussion

This mixed methods systematic review has identified overarching recovery themes (attributes) relevant to people who have undergone a TKR. It has found that pain, activities of daily living and mobility are the three most frequently mentioned attributes that are important to people undergoing TKR. These attribute list can be further developed, ideally with qualitative methodology to define a context-specific attribute set suitable for health preferencing techniques such as discrete choice experiments [12, 13].

For the practicing clinician, this review is a synthesis of evidence of recovery issues that people are considering a TKR value. This should form the basis of informed consent, providing an individualised discussion with each potential patient. A focus on major issues such as post-operative pain, mobility and return to activities of daily living has been identified from this review. Being aware of the range of issues, that may not be an individual greatest priority, but still a consideration, such as cosmetic appearance of the knee, should factor into clinical consultations to ensure ideas, concerns and expectation of patients can be explored thoroughly. Appropriately exploring such issues can give realistic expectations of recovery. This is crucial as unmet expectations are a source of dissatisfaction after TKR [19]. Health commissioners and systems should consider incorporating these findings into guidance given to surgeons for consent.

Pain, activities of daily living (ADL) and mobility are the three most reported themes in the literature, as found by this review. This is consistent with the literature, often being cited as the most common reasons people seek a TKR to improve [8, 41, 55]. Ongoing pain is particularly important and often highlighted as a strong determinant in satisfaction [3, 38, 42, 53]. There is a natural overlap between mobility and ADL, as many ADLs will require the ability to walk.

A less commonly reported theme was sexual function, which is underreported in the literature with respect to TKR [31]. Sexual function is not captured in commonly used PROMs such as the Oxford Knee Score, Forgotten Joint Score and Knee Society Score [4, 15, 47]. As a result, it is not posed as a recovery domain to participants in some studies, which may lead a reporting bias and it being an infrequently mentioned theme. Being an intimate topic, many patients may feel it is too sensitive of a topic to discuss during consultation [34], and therefore, its importance to an individual is often not established. Sexual function is limited pre-operatively by pain in 87%, of whom for 44% of people is due to a diminished range of motion of the knee joint [32]. Post-operatively, people were required to adjust their sexual position to accommodate their knee during sex [32]. A systematic review exploring return to sexual activity found polarised results, with some study studies reporting sexual activity, was amongst the most important, whilst other studies placed as amongst the least important [31]. It was the single most important activity out of 16, where importance was defined as a percentage of participants in each activity [31]. Comparatively, Scott et al. reported sexual function to be 16th of 17 variables by means of participants ranking importance by ‘very’ or ‘somewhat’ [55].

Szawlowski conducted a DCE asking surgeons to trade off patients potential improvement and risk of complications (not included in this review as assessing surgeon preferences) [56]. Whilst their methodology states they used a combination of systematic review, qualitative methods and expert opinion, the weight and the processes used to determine the final set of attributes are not fully reported. Nevertheless, they reported attributes of pain, functional outcomes and risk of complications, which compare well to the most frequently reported attributes in this systematic review.

### Strengths and limitations

This review provides an up-to-date summary of recovery attributes that are relevant to people recovering from a total knee replacement. The review methods were published prospectively and performed in accordance with PRISMA guidelines. It is an example of a rigorous and transparent method of how to conduct and report the initial development stages of attributes for a DCE. This provides the template methodology for future researchers conducting health economic research. Potential attributes derived from this review are from a large geographical spread, making this dataset generalisable across healthcare settings.

This study excluded eight studies (Fig. 1) which had mixed populations of lower limb or total joint arthroplasty. Whilst the authors of each study were contacted to see if raw data for TKR could be extracted, no response was received. This may reflect a publication bias within the final results, not fully accounting for some attributes and their relative preference. Other restrictions such as only English language may result in further publication bias to this review.

This mixed methods review did not determine preferences using the same method: whilst some studies conducted qualitative interviews, others used focus groups, or quantitative methods. Whilst some studies used statistical tests to compare between attributes, this was not possible in qualitative study designs. As a result, the heterogeneity in study designs has provided a broad stroke review, rather than granular insight into recovery preferences. Furthermore, studies did not examine the same set of attributes between people, which can make comparisons difficult. Some studies derived domains from PROM instruments, which authors deemed as important from the specific paper, rather than from patients themselves. Other papers generated these domains de novo using qualitative methods (such as interviews and focus groups).

This review details the frequency of attributes mentioned (giving the mode of each attribute) with limited insight into their preference against others. Quantitative studies provided weight on attributes, however, had little flexibility to introduce new themes or attributes as they frequently used PROM instruments with predefined attributes. Qualitative studies, whilst developing and introducing new attributes, were unable to provide relative weighting. Further development of reported themes with qualitative work can give context-specific insight into the importance of recovery attributes. These can be progressed to form attributes for a health preferencing study, such as a discrete choice experiment, which can gain relative weight of each attribute compared to each another, providing granular level importance of one recovery domain compared to another.

## Conclusion

This systematic review contributes to the literature by synthesising current evidence of recovery preferences after TKR. It has found the most reported themes for recovery were reduction in pain, return to activities of daily living, return to mobility and return to recreational sports and activities. Clinicians should be aware of these themes as potential motivators for surgery and ensure they are addressed when people are considering TKR. For health preference researchers, these data can form the basis to produce context-specific attribute development in experimental designs such as discrete choice experiments.

### Supplementary Information

Below is the link to the electronic supplementary material.

Supplementary file1 (DOCX 15 KB)

Supplementary file2 (DOCX 18 KB). Mixed Methods Appraisal Tool description of included studies.

Supplementary file3 (DOCX 39 KB)

## Data Availability

All data available is presented in this manuscript and supplementary materials.

## References

[1] Anderson JG, Wixson RL, Tsai D, Stulberg SD, Chang RW (1996). Functional outcome and patient satisfaction in total knee patients over the age of 75. J Arthroplasty.

[2] Ayers DC, Yousef M, Zheng H, Yang W, Franklin PD (2022). The prevalence and predictors of patient dissatisfaction 5-years following primary total knee arthroplasty. J Arthroplasty.

[3] Baker PN, van der Meulen JH, Lewsey J, Gregg PJ (2007). The role of pain and function in determining patient satisfaction after total knee replacement. Data from the National Joint Registry for England and Wales. J Bone Joint Surg.

[4] Behrend H, Giesinger K, Giesinger JM, Kuster MS (2012). The “forgotten joint” as the ultimate goal in joint arthroplasty: validation of a new patient-reported outcome measure. J Arthroplasty.

[5] Bellamy N, Buchanan WW, Goldsmith CH, Campbell J, Stitt LW (1988). Validation study of WOMAC: a health status instrument for measuring clinically important patient relevant outcomes to antirheumatic drug therapy in patients with osteoarthritis of the hip or knee. J Rheumatol.

[6] Ben-Shlomo Y, Blom A, Boulton C, et al. (2022) The National Joint Registry 19th Annual Report 2022. 36516281

[7] Bin Sheeha B, Williams A, Granat M, Jones R, Johnson DS (2020). Patients' experiences and satisfaction at one year following primary total knee arthroplasty: A focus-group discussion. Musculoskelet Care.

[8] Bourne RB, Chesworth BM, Davis AM, Mahomed NN, Charron KD (2010). Patient satisfaction after total knee arthroplasty: who is satisfied and who is not?. Clin Orthop Relat Res.

[9] Braun V, Clarke V (2006). Using thematic analysis in psychology. Qual Res Psychol.

[10] Chan EY, Blyth FM, Nairn L, Fransen M (2013). Acute postoperative pain following hospital discharge after total knee arthroplasty. Osteoarthr age.

[11] Cheow S-L, Chan E-Y, Fransen M, Blyth FM (2013). Postoperative pain following hospital discharge after knee replacement surgery: a patient survey. Pain Manag.

[12] Coast J, Al-Janabi H, Sutton EJ (2012). Using qualitative methods for attribute development for discrete choice experiments: issues and recommendations. Health Econ.

[13] Coast J, Horrocks S (2007). Developing attributes and levels for discrete choice experiments using qualitative methods. J Health Serv Res Policy.

[14] Cross M, Lapsley H, Barcenilla A, Parker D, Coolican M, March L (2009). Patient expectations of hip and knee joint replacement surgery and postoperative health status. Patient.

[15] Dawson J, Fitzpatrick M, Churchman D, Verjee-Lorenz A, Claysonm D (2010). User manual for the Oxford Knee Score (OKS).

[16] de Achaval S, Kallen MA, Zhang H (2016). Patients' expectations about total knee arthroplasty outcomes. Health Expect.

[17] DeFrance M, Scuderi G (2023). Are 20% of Patients actually dissatisfied following total knee arthroplasty? A systematic review of the literature. J Arthroplasty.

[18] Devers BN, Noble PC (2011). Does greater knee flexion increase patient function and satisfaction after total knee arthroplasty?. J Arthroplasty.

[19] Dunbar MJ, Richardson G, Robertsson O (2013). I can’t get no satisfaction after my total knee replacement: rhymes and reasons. Bone Joint J.

[20] E JS, Soon VL, Boyd A, McAllister J, Deakin AH, Sarungi M (2016). What do scottish patients expect of their total knee arthroplasty?. J Arthroplasty.

[21] Espinosa A, Jimenez M, Zorrilla P, Lopez A, Salido JA, Amo M (2020). Influence of fulfilment patient expectations in outcomes after total knee arthroplasty. Influencia del cumplimiento de las expectativas del paciente en los resultados de la artroplastia total de rodilla.

[22] Griffin J, Davis ET, Parsons H (2023). Robotic arthroplasty clinical and cost effectiveness randomised controlled trial (RACER-knee): a study protocol. BMJ Open.

[23] Harmsen RTE, Haanstra TM, Den Oudsten BL (2020). A high proportion of patients have unfulfilled sexual expectations after TKR: a prospective study. Clin Orthop Relat Res.

[24] Health NIo (2003). NIH Consensus Statement on total knee replacement. NIH Consens State Sci Statements.

[25] Helter TM, Boehler CEH (2016). Developing attributes for discrete choice experiments in health: a systematic literature review and case study of alcohol misuse interventions. Journal of substance use.

[26] Hong QN, Fàbregues S, Bartlett G (2018). The Mixed Methods Appraisal Tool (MMAT) version 2018 for information professionals and researchers. Educ Inf.

[27] Hutyra CA, Gonzalez JM, Yang J-C (2020). Patient preferences for surgical treatment of knee osteoarthritis: a discrete-choice experiment evaluating total and unicompartmental knee arthroplasty. JBJS.

[28] Indicators O (2019). Health at a glance 2019: OECD indicators.

[29] Jeffery AE, Wylde V, Blom AW, Horwood JP (2011). "it's there and I'm stuck with it": Patients' experiences of chronic pain following total knee replacement surgery. Arthritis Care Res.

[30] Kahlenberg CA, Nwachukwu BU, McLawhorn AS, Cross MB, Cornell CN, Padgett DE (2018). Patient satisfaction after total knee replacement: a systematic review. HSS J.

[31] Kazarian GS, Chen AF (2017). Patients experience mixed results with respect to sexual quality and frequency after total knee arthroplasty: a systematic review. J ISAKOS.

[32] Kazarian GS, Lonner JH, Hozack WJ, Woodward L, Chen AF (2017). Improvements in sexual activity after total knee arthroplasty. J Arthroplasty.

[33] Khatri C, Ahmed I, Parsons H (2019). The natural history of full-thickness rotator cuff tears in randomized controlled trials: a systematic review and meta-analysis. Am J Sports Med.

[34] Klit J, Jacobsen S, Rosenlund S, Sonne-Holm S, Troelsen A (2014). Total knee arthroplasty in younger patients evaluated by alternative outcome measures. J Arthroplasty.

[35] Kwoh CK, Vina ER, Cloonan YK, Hannon MJ, Boudreau RM, Ibrahim SA (2015). Determinants of patient preferences for total knee replacement: African-Americans and whites. Arthritis Res Ther.

[36] Leventhal HR, Ian B (2012). The common-sense model of self-regulation of health and illnessThe self-regulation of health and illness behaviour.

[37] Lewis S, Price M, Dwyer KA (2014). Development of a scale to assess performance following primary total knee arthroplasty. Value Health.

[38] Lingard EA, Sledge CB, Learmonth ID (2006). Patient expectations regarding total knee arthroplasty: Differences among the United States, United Kingdom, and Australia. J Bone Joint Surg Ser A.

[39] Macario A, Schilling P, Rubio R, Bhalla A, Goodman S (2003). What questions do patients undergoing lower extremity joint replacement surgery have?. BMC Health Serv Res.

[40] Mahdi A, Svantesson M, Wretenberg P, Halleberg-Nyman M (2020). Patients' experiences of discontentment one year after total knee arthroplasty- a qualitative study. BMC Musculoskelet Disord.

[41] Mancuso CA, Sculco TP, Wickiewicz TL (2001). Patients' expectations of knee surgery. J Bone Joint Surg Am.

[42] Mannion AF, Kampfen S, Munzinger U, Kramers-de Quervain I (2009). The role of patient expectations in predicting outcome after total knee arthroplasty. Arthritis Res Ther.

[43] Mavalankar AP, Rani S (2019). Is achieving high flexion necessary for satisfaction after total knee arthroplasty in Indian patients. Indian J Orthopaed.

[44] McGrory BJ, Berry DJ, Becker MW, Harmsen WS, Trousdale RT (1999). Patients' concerns prior to undergoing total hip and total knee arthroplasty. Mayo Clin Proc.

[45] Muertizha M, Cai X, Ji B, Aimaiti A, Cao L (2022). Factors contributing to 1-year dissatisfaction after total knee arthroplasty: a nomogram prediction model. J Orthop Surg Res.

[46] Muniesa JM, Marco E, Tejero M (2010). Analysis of the expectations of elderly patients before undergoing total knee replacement. Arch Gerontol Geriatr.

[47] Noble PC, Scuderi GR, Brekke AC (2012). Development of a new Knee Society scoring system. Clin Orthop Relat Res.

[48] Obadha M, Barasa E, Kazungu J, Abiiro GA, Chuma J (2019). Attribute development and level selection for a discrete choice experiment to elicit the preferences of health care providers for capitation payment mechanism in Kenya. Heal Econ Rev.

[49] Page MJ, McKenzie JE, Bossuyt PM (2021). The PRISMA 2020 statement: an updated guideline for reporting systematic reviews. BMJ.

[50] Robertsson O, Dunbar M, Pehrsson T, Knutson K, Lidgren L (2000). Patient satisfaction after knee arthroplasty: a report on 27,372 knees operated on between 1981 and 1995 in Sweden. Acta Orthop Scand.

[51] Ryan M, Gerard K, Amaya-Amaya M (2007). Using discrete choice experiments to value health and health care.

[52] Scotland PH (2021) Scottish Arthroplasty Project National Report 2021.

[53] Scott C, Howie C, MacDonald D, Biant L (2010). Predicting dissatisfaction following total knee replacement: a prospective study of 1217 patients. J Bone Joint Surg.

[54] Scott CE, Oliver WM, MacDonald D, Wade FA, Moran M, Breusch SJ (2016). Predicting dissatisfaction following total knee arthroplasty in patients under 55 years of age. Bone Joint J.

[55] Scott CEH, Bugler KE, Clement ND, MacDonald D, Howie CR, Biant LC (2012). Patient expectations of arthroplasty of the hip and knee. J Bone Joint Surg.

[56] Szawlowski S, Choong PF, Li J (2019). How do surgeons’ trade-off between patient outcomes and risk of complications in total knee arthroplasty a discrete choice experiment in Australia. BMJ Open.

[57] Wiering B, de Boer D, Delnoij D (2017). Asking what matters: The relevance and use of patient-reported outcome measures that were developed without patient involvement. Health Expect.

