# Supplementary material for: What’s important for recovery after a total knee replacement? A systematic review of mixed methods studies
Source: Arch Orthop Trauma Surg. 2023 Dec 9;144(5):2213–21. doi: 10.1007/s00402-023-05136-x (PMC11093842; doi:10.1007/s00402-023-05136-x)
Supplement: Supplementary file 1 — Supplementary file1 (DOCX 15 KB) [file 402_2023_5136_MOESM1_ESM.docx]

**Ovid, MEDLINE(R) & PyschInfo ALL <1946 to December 1, 2021>**

1 knee arthroplasty.mp. or exp Arthroplasty, Replacement, Knee/

2 (TKR or TKA or Total knee replacement or knee replacement).mp. [mp=title, abstract, original title, name of substance word, subject heading word, floating sub-heading word, keyword heading word, organism supplementary concept word, protocol supplementary concept word, rare disease supplementary concept word, unique identifier, synonyms]

3 1 or 2

4 exp "Recovery of Function"/

5 Recover*.mp.

6 exp Rehabilitation/

7 4 or 5 or 6

8 3 and 7

9 (patient adj5 (opinion* or aThe ttitude* or satisfy*)).mp. [mp=title, abstract, original title, name of substance word, subject heading word, floating sub-heading word, keyword heading word, organism supplementary concept word, protocol supplementary concept word, rare disease supplementary concept word, unique identifier, synonyms]

10 8 and 9

11 (Patient adj5 (prefer* or choice or choice experiment* or choice behaviour* or choice behavior*)).mp. [mp=title, abstract, original title, name of substance word, subject heading word, floating sub-heading word, keyword heading word, organism supplementary concept word, protocol supplementary concept word, rare disease supplementary concept word, unique identifier, synonyms]

12 3 and 11

13 12 or 10

**Cochrane**

ID Search Hits

#1 MeSH descriptor: [Arthroplasty, Replacement, Knee] explode all trees

#2 MeSH descriptor: [Recovery of Function] explode all trees

#3 MeSH descriptor: [Postoperative Care] explode all trees

#4 #2 OR #3

#5 #1 AND #4

#6 MeSH descriptor: [Patient Satisfaction] 3 tree(s) exploded

#7 #1 AND #6

#8 MeSH descriptor: [Patient Preference] explode all trees

#9 #1 AND #8

#10 #5 OR #7 or #9

**Web of Science**

1

(((((((((TS=(“knee arthroplasty”)) OR TS=(“knee replacement”)) OR TS=(“tkr”)) OR TS=(“TKA”)) OR TS =(“total knee replacement”) OR TS=(“Total knee arthroplasty”))))))

2

((TS=("patient satisfaction")) OR TS=("Patient opinion*")) OR TS=("patient attitude*")

3

(TS=(Recovery)) OR TS=(Rehab*)

Final search = #1 AND #2 AND #3
