# Supplementary material for: What’s important for recovery after a total knee replacement? A systematic review of mixed methods studies
Source: Arch Orthop Trauma Surg. 2023 Dec 9;144(5):2213–21. doi: 10.1007/s00402-023-05136-x (PMC11093842; doi:10.1007/s00402-023-05136-x)
Supplement: Supplementary file 2 — Supplementary file2 (DOCX 18 KB). Mixed Methods Appraisal Tool description of included studies. [file 402_2023_5136_MOESM2_ESM.docx]

|  | **Qualitative Studies** | | | | |
| --- | --- | --- | --- | --- | --- |
|  | 1.1. Is the qualitative approach appropriate to answer the research question? | 1.2. Are the qualitative data collection methods adequate to address the research question? | 1.3. Are the findings adequately derived from the data? | 1.4. Is the interpretation of results sufficiently substantiated by data? | 1.5. Is there coherence between qualitative data sources, collection, analysis and interpretation? |
| Bin Sheeha | Yes | Yes | Yes | Yes | Yes |
| Jeffrey | Yes | Yes | Yes | Yes | Yes |
| Mahdi | Yes | Yes | Yes | Yes | Yes |
| Lewis | Yes | Yes | Yes | Yes | Yes |
|  | **Quantitative Descriptive Studies** | | | | |
|  | 4.1. Is the sampling strategy relevant to address the research question? | 4.2. Is the sample representative of the target population? | 4.3. Are the measurements appropriate? | 4.4. Is the risk of nonresponse bias low? | 4.5. Is the statistical analysis appropriate to answer the research question? |
| Cross | Yes | Yes | Yes | No | Yes |
| Chan | Yes | Yes | Yes | Yes | Yes |
| Cheow | Yes | Yes | Yes | Yes | No |
| De Achaval | Yes | Yes | Yes | Yes | Yes |
| Devers | Yes | Yes | Yes | Can't tell | Yes |
| Espinosa | Can't tell | Can't tell | Yes | Can't tell | Yes |
| Harmsen | Yes | Yes | Yes | No | Yes |
| Hutyra | Yes | Yes | Yes | Yes | Yes |
| Lingard | Yes | Yes | Yes | Yes | Yes |
| Mannion | Yes | Yes | Yes | No | Yes |
| Mavalankar | Yes | Yes | Yes | No | No |
| McGrory | Yes | Can't tell | Yes | Can't tell | Yes |
| Muniesa | Yes | Yes | Yes | Yes | Yes |
| Noble | Yes | Can’t Tell | Yes | Can’t tell | Yes |
| Scott | Yes | Yes | Yes | Yes | Yes |
| Wiering | Yes | Yes | Yes | Yes | Yes |
| Kwoh | Yes | Yes | Yes | Can't tell | Yes |
| Smith | Yes | Yes | Yes | Yes | Yes |
|  | **Mixed Methods** | | | | |
|  | 5.1. Is there an adequate rationale for using a mixed methods design to address the research question? | 5.2. Are the different components of the study effectively integrated to answer the research question? | 5.3. Are the outputs of the integration of qualitative and quantitative components adequately interpreted? | 5.4. Are divergences and inconsistencies between quantitative and qualitative results adequately addressed? | 5.5. Do the different components of the study adhere to the quality criteria of each tradition of the methods involved? |
| Macario | Yes | Yes | Yes | Can't tell | Yes |

**Supplementary File 2: Mixed Methods Appraisal Tool description of included studies**
