# Supplementary material for: What’s important for recovery after a total knee replacement? A systematic review of mixed methods studies
Source: Arch Orthop Trauma Surg. 2023 Dec 9;144(5):2213–21. doi: 10.1007/s00402-023-05136-x (PMC11093842; doi:10.1007/s00402-023-05136-x)
Supplement: Supplementary file 3 — Supplementary file3 (DOCX 39 KB) [file 402_2023_5136_MOESM3_ESM.docx]

| **Overarching Theme** | **Attribute listed in study** | **Importance** | **Level/Modality** | **Author** |
| --- | --- | --- | --- | --- |
| Pain | Relieve pain | Mean score of 2.7 (3.0 = very important) |  | Cross |
|  | Post-operative pain |  | 2-week period post-op | Chan |
|  | Post-operative pain | 40% reported frequent, severe pain  20% reported this is the most painful period | 2-week period post-op | Cheow |
|  | Improvement in pain | 88.5% expected by 1 year post-operatively |  | Espinosa |
|  | Dealing with chronic pain |  |  | Jeffrey |
|  | Pain | 71.9% expected to have no pain at 12 months | None, slight, moderate, severe | Lingard |
|  | Will surgery cause pain after | Scored 4 (5 =most important) |  | Macario |
|  | Pain |  | 6 weeks, 3 months, 1 year | Mahdi |
|  | Pain at rest |  | 6 weeks, 3 months, 1 year | Mahdi |
|  | Pain at night |  | 6 weeks, 3 months, 1 year | Mahdi |
|  | Pain | 85% expected no knee pain | 2 years | Mannion |
|  | Pain relief by operation | 68/69 found pain relief from the operation |  | Mavalankar |
|  | Pain immediately after surgery | Mean level of concern was (2.03), joint third most important in this paper |  | McGrory |
|  | Pain | 99.80% expected relief |  | Muniesa |
|  | Relieve pain | Ranked 1st |  | Smith |
|  | Relieve pain in the joint that interferes with sleep | 70% said very important |  | Scott |
|  | Relieve daytime pain in joint | 85% said very important |  | Scott |
|  | Pain at rest | 66.5% said very important |  | Wiering |
|  | Pain during use | 61.1% said very important |  | Wiering |
|  | Less post-op surgical pain | Not weighted against other attributes |  | Kwoh |
|  | | | | |
| Activities of Daily Living | Improvement in ADL |  | Walking, ability to kneel, stairs | Bin Sheeha |
|  | Improve ability to go up stairs | Mean score of 2.7 (3.0 = very important) |  | Cross |
|  | Improve ability to go down stairs | Mean score of 2.7 (3.0 = very important) |  | Cross |
|  | Improve ability to perform daily activities | Mean score of 2.7 (3.0 = very important) |  | Cross |
|  | Improve ability to change position | Mean score of 2.6 (3.0 = very important) |  | Cross |
|  | Improve ability to use public transport | Mean score of 2.7 (3.0 = very important) |  | Cross |
|  | Getting in/out of car | Ranked 2^nd^ | 1 week, 6 weeks, 3 months, 6 months | De Achaval |
|  | Put on socks | Ranked 3^rd^ | 1 week, 6 weeks, 3 months, 6 months | De Achaval |
|  | Drive | Ranked 4^th^ | 1 week, 6 weeks, 3 months, 6 months | De Achaval |
|  | Perform light domestic duties | Ranked 5^th^ | 1 week, 6 weeks, 3 months, 6 months | De Achaval |
|  | Go up and down stairs | Ranked 6^th^ | 1 week, 6 weeks, 3 months, 6 months | De Achaval |
|  | Go shopping | Ranked 7^th^ | 1 week, 6 weeks, 3 months, 6 months | De Achaval |
|  | Improvement in daily life activities | 35.% expected this by 1 year post-operatively | 1 year | Espinosa |
|  | Improvement in walking up stairs | 12.6% expected this by 1 year post-operatively | 1 year | Espinosa |
|  | Improvement in social activities | 2.7% expected this by 1 year post-operatively | 1 year | Espinosa |
|  | Improvement in going down stairs | 1.1% expected this by 1 year post-operatively | 1 year | Espinosa |
|  | Improvement in using the bus or metro | 0% expected this by 1 year post-operatively | 1 year | Espinosa |
|  | Function | Ranked 4^th^ | Lead active lifestyle, do all activities of daily living, do activity of daily living with some limitations | Hutyra |
|  | Modification | Not weighted against other attributes | Less likely to use stairs, putting on socks | Lewis |
|  | Inability to do activities of daily living | Wash dishes, vacuum cleaning, cutting the grass |  | Mahdi |
|  | Will the surgery affect my abilities to care for myself | Scored 5 (5 =most important) |  | Macario |
|  | How will I be able to bathe myself after the surgery | Scored 4 (5 =most important) |  | Macario |
|  | Will I be able to do the household chores/work in the garden | Scored 3.5 (5 =most important) |  | Macario |
|  | Will the surgery affect the way I sleep | Scored 4 (5 =most important) |  | Macario |
|  | Inability to do activities of daily living | Wash dishes, vacuum cleaning, cutting the grass |  | Mahdi |
|  | Ability to go up and down stairs | Mean level of concern was (2.04), second most important in this paper |  | McGrory |
|  | Public transport | 88.70% expected this |  | Muniesa |
|  | Going up stairs | 99.60% expected this |  | Muniesa |
|  | Going down stairs | 99.60% expected this |  | Muniesa |
|  | Gardening | 14% said this was the most important activity |  | Noble |
|  | Improve ability to perform daily activities away from the home | 64% said very important |  | Scott |
|  | Improve ability to perform daily activities around the home | 72% said very important |  | Scott |
|  | Improve ability to get in or out of a bed, chair, car or bus | 75% said very important |  | Scott |
|  | Improve ability to climb stairs | 77% said very important |  | Scott |
|  | Improve ability to go down stairs | 78% said very important |  | Scott |
|  | Improve ability to go down stairs | Ranked 3rd |  | Smith |
|  | Improve ability to go up stairs | Ranked 4th |  | Smith |
|  | Improve ability to perform daily activities | Ranked 5th |  | Smith |
|  | Improve ability to change positions | Ranked 6th |  | Smith |
|  | Improve ability to use public transportation or drive | Ranked 7th |  | Smith |
|  | Rising from bed | 40.5% said very important |  | Wiering |
|  | Putting on socks | 36.1% said very important |  | Wiering |
|  | | | | |
| Mobility (Walking) | Daily time spent walking |  | <15 min, 15-30, 31-60, >60 min | Chan |
|  | Daily time spent walking | 59% of participants walked more than 30 mins | >30 minutes | Cheow |
|  | Improve ability to walk | Mean score of 2.75 (3.0 = very important) |  | Cross |
|  | Improvement in mobility | 2.2% expected this by 1 year post-operatively |  | Espinosa |
|  | Natural motion confidence | Not weighted against other attributes | Walking on slippery surface, kneeling on knee, getting down on a toilet, walking on uneven surface | Lewis |
|  | Distance able to walk without support | 38.3% expected to be able to walk max distance at 12 months | >2miles, 1-2 miles, about 0.5miles, few yards | Lingard |
|  | How mobile will I be after my surgery | Scored 5 (5 =most important) |  | Macario |
|  | When will I be able to walk normally again | Scored 5 (5 =most important) |  | Macario |
|  | Walking | Walk 100-250m | 1 year | Mahdi |
|  | Ability to walk as much as you wish | Mean level of concern was (2.08), most important in this paper |  | McGrory |
|  | Walking | 100% expected this |  | Muniesa |
|  | Everyday activities (walking) | 93% expected this |  | Muniesa |
|  | Distance walking | 19% said this was the most important activity |  | Noble |
|  | Less walking difficulties | Not weighted against other attributes |  | Kwoh |
|  | Improve ability to walk | 92% said very important |  | Scott |
|  | Improve ability to walk | Ranked 2nd |  | Smith |
|  | | | | |
| Recreational Activities and Sport | Improve ability to participate in recreational activity | Mean score of 2.6 (3.0 = very important) |  | Cross |
|  | Improve ability to exercise/play sport | Mean score of 2.3 (3.0 = very important) |  | Cross |
|  | Improve ability to interact with others | Mean score of 2.3 (3.0 = very important) |  | Cross |
|  | Improvement for leisure activities | 10.4% expected this by 1 year post-operatively |  | Espinosa |
|  | Improvement in playing sport | 0.5% expected this by 1 year post-operatively |  | Espinsoa |
|  | Recreational limitations | 33.6% expected no recreational limitations at 12 months | None, slight, moderate, extreme | Lingard |
|  | Will my surgery decision affect my social life | Scored 3.5 (5 =most important) |  | Macario |
|  | Recreational activities | e.g., biking, dancing, fishing, playing golf, skiing, swimming |  | Mahdi |
|  | Leisure activities | 86.90% expected this |  | Muniesa |
|  | Sport | 22.90% expected this |  | Muniesa |
|  | Social activities | 92.40% expected this |  | Muniesa |
|  | Stretching | 11.50% said this was the most important activity |  | Noble |
|  | Swimming | 10% said this was the most important activity |  | Noble |
|  | Stationary Bike | 9% said this was the most important activity |  | Noble |
|  | Golf | 7.50% said this was the most important activity |  | Noble |
|  | Road cycling | 4% said this was the most important activity |  | Noble |
|  | Dancing | 4% said this was the most important activity |  | Noble |
|  | Stair climber | 3.50% said this was the most important activity |  | Noble |
|  | Leg extension | 3% said this was the most important activity |  | Noble |
|  | Aerobic exercises | 3% said this was the most important activity |  | Noble |
|  | Weightlifting | 3% said this was the most important activity |  | Noble |
|  | Elliptical trainer | 2% said this was the most important activity |  | Noble |
|  | Racquet sports | 1.50% said this was the most important activity |  | Noble |
|  | Bowling | 1% said this was the most important activity |  | Noble |
|  | Leg press | 1% said this was the most important activity |  | Noble |
|  | Jogging | 0.50% said this was the most important activity |  | Noble |
|  | Improve ability to participate in recreation or social activities | 45% said very important |  | Scott |
|  | Improve ability to exercise or take part in recreational sports | 48% said very important |  | Scott |
|  | Improve ability to participate in recreational activities | Ranked 9th |  | Smith |
|  | Improve ability to interact with others | Ranked 10th |  | Smith |
|  | Improve ability to exercise or participate in sports | Ranked 14th |  | Smith |
|  | | | | |
| Specific Functional Movements | Improve ability to kneel | Mean score of 2.3 (3.0 = very important) |  | Cross |
|  | Improve ability to squat | Mean score of 2.25 (3.0 = very important) |  | Cross |
|  | Rise from sitting | Ranked 1^st^ | 1 week, 6 weeks, 3 months, 6 months | De Achaval |
|  | Kneel | Ranked 9^th^ | 1 week, 6 weeks, 3 months, 6 months | De Achaval |
|  | Improvement in kneeling | 2.2% expected this by 1 year post-operatively |  | Espinosa |
|  | Improvement in squatting | 1.1% expected this by 1 year post-operatively |  | Espinosa |
|  | Sitting cross legged |  |  | Mavanlanker |
|  | Kneeling | 50.50% expected this |  | Muniesa |
|  | Squatting | 41.90% expected this |  | Muniesa |
|  | Improve ability to squat | 52% said very important |  | Scott |
|  | Improve ability to kneel | 63% said very important |  | Scott |
|  | Improve ability to kneel | Ranked 11^th^ |  | Smith |
|  | Improve ability to squat | Ranked 15^th^ |  | Smith |
|  | Bending to the floor | 32.6% said very important |  | Wiering |
|  | Twisting/pivoting on your injured knee | 26.6% said very important |  | Wiering |
|  | Kneeling | 21.2% said very important |  | Wiering |
|  | Squatting | 19.4% said very important |  | Wiering |
|  | Rising from sitting | 41.3% said very important |  | Wiering |
|  | | | | |
| Use of Walking Aids | Remove need for crutch | Mean score of 2.6 (3.0 = very important) |  | Cross |
|  | Walk 1 block without cane | Ranked 8^th^ | 1 week, 6 weeks, 3 months, 6 months | De Achaval |
|  | Walk 5 blocks without cane | Ranked 10^th^ | 1 week, 6 weeks, 3 months, 6 months | De Achaval |
|  | Does not need crutch or stick | 20.2% expected this by 1 year post-operatively |  | Espinosa |
|  | Walking aid required | 85.9% expected no walking aid | None, stick/cane, crutches, walker | Lingard |
|  | Use of walking aid | Did not affect satisfaction |  | Mavanlankar |
|  | Not using a cane | 46.30% expected this |  | Muniesa |
|  | Remove the need for a stick | 56% said very important |  | Scott |
|  | Remove the need for cane, crutch or walker | Ranked 13^th^ |  | Smith |
|  | | | | |
| Range of Motion of the Knee | Make knee straight | Mean score of 2.6 (3.0 = very important) |  | Cross |
|  | Knee flexion | Increased knee flexion had significant positive association with achievement of expectation | Low (<110), mid (111-130, high >130) | Devers |
|  | Motion | Not weighted against other attributes | Twisting or pivoting | Lewis |
|  | Satisfaction | Not weighted against other attributes | Bending | Lewis |
|  | Stiffness (lack of bending) | To be able to behind the knee as normal |  | Mahdi |
|  | Knee flexion | Did not affect satisfaction |  | Mavalankar |
|  | Mobility of the knee | 100% expected this |  | Muniesa |
|  | Make knee or leg straight | 57% said very important |  | Scott |
|  | Make knee or leg straight | Ranked 12^th^ |  | Smith |
|  | | | | |
| Sexual Activity | Improve sexual activity | Mean score of 2.2 (3.0 = very important) |  | Cross |
|  | Improvement in sexual activities | 0.5% expected this by 1 year post-operatively |  | Espinosa |
|  | Sexual function | 54% expected return to sexual function by 12 months post-operatively | Back to normal, Large improvement, Moderate improvement, Slight improvement | Hamsen |
|  | Will the surgery affect my sexual functioning | Scored 2 (5 =most important) |  | Macario |
|  | Sexual activities | 4.80% expected this |  | Muniesa |
|  | Sexual activity | 16% said very important |  | Scott |
|  | Improve sexual activity | Ranked 16^th^ |  | Smith |
|  | | | | |
| Psychological Well Being | Improve psychological well being | Mean score of 2.6 (3.0 = very important) |  | Cross |
|  | Improvement in sensation of well being | 9.8% expected this by 1 year post-operatively |  | Espinosa |
|  | General well being | 99.80% expected this |  | Muniesa |
|  | Improve psychological well-being | 58% said very important |  | Scott |
|  | Improve psychological well being | Ranked 8^th^ |  | Smith |
|  | | | | |
| Employment | Employed for monetary reimbursement | Mean score of 2.3 (3.0 = very important) |  | Cross |
|  | Ability to work | 2.7% expected this by 1 year post-operatively | 1 year | Espinosa |
|  | Paid employment | 5% expected this |  | Muniesa |
|  | Employed for monetary reimbursement | 15% said very important |  | Scott |
|  | Be employed for monetary reimbursement | Ranked 17^th^ |  | Smith |
|  | | | | |
| Forgottenness of Joint Replacement | Forgottenness of joint | No relative importance given |  | Bin Sheeha |
|  | Normality | Ranked 3^rd^ | Never-, Seldom-, Mostly- aware of knee joint | Hutyra |
|  | Forgotten knee | Not weighted against other attributes | Whilst doing activities: Kneeling, going up and down stairs, stepping on a ladder, squatting, riding in a vehicle, walking on concrete, bending on hands and knees, wedding, getting out of bed, remaining in the same position | Lewis |
|  | | | | |
| Medical Complications | Serious complications | Ranked 1^st^ | 1,4,11% | Hutyra |
|  | Revision rates | Ranked 2^nd^ | 3,12,19% | Hutyra |
|  | Will I have an increased chance of bleeding after surgery | Scored 4 (5 =most important) |  | Macario |
|  | Risk of getting AIDS from transfusion | Mean level of concern was (1.99), fifth most important in this paper |  | McGrory |
|  | | | | |
| Timing of Recovery | Time to recovery | Time to expected full recovery was 4.7 months | 5 months | Mannion |
|  | Return to normal function | 52% expected no limitations |  | Mannion |
|  | Length of recovery | Mean level of concern was (2.03), joint third most important in this paper |  | McGrory |
|  | | | | |
| Cosmetic Appearance | Will the surgery change the way I look | Scored 2 (5 =most important) |  | Macario |
|  | Cosmetic appearance of the knee | Felt knee was 'fat' and swollen |  | Mahdi |
|  | | | | |
| Length of stay | How long will I be in the hospital | Scored 4 (5 =most important) |  | Macario |
|  | Shorter hospital course | Not weighted against other attributes |  | Kwoh |
|  | | | | |
| Stability | Stability | Not weighted against other attributes | Feeling of going to pop | Lewis |
|  | | | | |
| Overall Experience | Overall recovery experience | No relative importance given |  | Bin Sheeha |
|  | | | | |
| Need for Physical Therapy | Am I going to need physical therapy | Scored 5 (5 =most important) |  | Macario |
